# Elevated expression of macrophage MERTK exhibits profibrotic effects and results in defective regulation of efferocytosis function in pulmonary fibrosis

**DOI:** 10.1186/s12931-023-02424-3

**Published:** 2023-04-29

**Authors:** Yixin She, Xin Xu, Qingyang Yu, Xiangsheng Yang, Jianxing He, Xiao Xiao Tang

**Affiliations:** 1 State Key Laboratory of Respiratory Disease, National Clinical Research Center for Respiratory Disease, National Center for Respiratory Medicine, Guangzhou Institute of Respiratory Health, The First Affiliated Hospital of Guangzhou Medical University, Guangzhou, China; 2 Guangzhou Laboratory, Bio-Island, Guangzhou, China

## Abstract

Increased apoptosis of alveolar epithelial cells is a prominent feature of pulmonary fibrosis. Macrophage efferocytosis, phagocytosis of apoptotic cells by macrophages, is crucial for maintaining tissue homeostasis. Expression of Mer tyrosine kinase (MERTK, an important recognition receptor in efferocytosis) in macrophages is thought to be associated with fibrosis. However, how macrophage MERTK affects pulmonary fibrosis and whether it depends on efferocytosis are not yet clear. Here, we found elevated MERTK expression in lung macrophages from IPF patients and mice with bleomycin-induced pulmonary fibrosis. In vitro experiments showed that macrophages overexpressing MERTK exhibit profibrotic effects and that macrophage efferocytosis abrogates the profibrotic effect of MERTK by downregulating MERTK, forming a negative regulatory loop. In pulmonary fibrosis, this negative regulation is defective, and MERTK mainly exhibits profibrotic effects. Our study reveals a previously unsuspected profibrotic effect of elevated macrophage MERTK in pulmonary fibrosis and defective regulation of efferocytosis function as a result of that elevation, suggesting that targeting MERTK in macrophages may help to attenuate pulmonary fibrosis.

**Supplementary Information:**

The online version contains supplementary material available at 10.1186/s12931-023-02424-3.

## Introduction

Idiopathic pulmonary fibrosis (IPF), the most common type of pulmonary fibrosis, is a fibrotic lung disease of unknown cause [1]. It is characterized by the accumulation of damaged and apoptotic alveolar epithelial cells, excessive proliferation and activation of fibroblasts, deposition of massive extracellular matrix, and infiltration of inflammatory cells [1–4]. The progression of IPF is irreversible [5]. Due to the complex aetiology and unclear pathogenesis, there are presently few effective targeted therapeutics in the clinic. Therefore, it is essential to elucidate the pathogenesis of IPF and identify therapeutic targets.

Massive damage and apoptosis of alveolar epithelial cells are features of IPF patients and mice with bleomycin-induced pulmonary fibrosis [3, 6, 7]. A study by Morimoto et al. showed defective alveolar macrophage efferocytosis in IPF patients compared to patients with other interstitial lung diseases [8]. However, whether the macrophage efferocytosis function in IPF is different from that in healthy controls is unclear.

Efferocytosis refers to phagocytosis of apoptotic cells, mostly by macrophages and to a lesser extent by monocytes, dendritic cells, and epithelial cells [9]. Efferocytosis plays an important role in preventing inflammation and maintaining tissue homeostasis [10]. The process of macrophage efferocytosis includes the following: (1) apoptotic cells release soluble signals (such as chemokines) to attract macrophages and stimulate their scavenging potential; (2) receptors (such as MERTK, CD36, etc.) on the surface of macrophages regulate indirect or direct recognition of apoptotic cells [11]; (3) Rac1 promotes uptake of apoptotic cells by regulating actin polymerization and pseudopodia extension [12]; (4) uncoupling protein 2 (Ucp2) plays an important role in lowering mitochondrial membrane potential to promote continued uptake and clearance of apoptotic cells [13]; and (5) the phagosome fuses with lysosomes and becomes a phagolysosome. The phagosome contents are then degraded and the newly formed phagolysosome can be defined by its high acidity (pH 4.5–5.0) and active cathepsins [9]. As a consequence of efferocytosis, the production of 15-LO (15-lipoxygenase) and IL-10 by macrophages prevents the induction of autoimmunity and permanent tissue inflammation [11, 14, 15].

Mer tyrosine kinase (MERTK), a member of the Tyro-3, Axl, and Mer (TAM) receptor tyrosine kinase family, is the main apoptotic cell receptor on macrophages [11]. Morse et al. found elevated macrophage MERTK expression in the lung tissue lesions of IPF patients compared to nonfibrotic lesions [16]. In nonalcoholic steatohepatitis, macrophage MERTK promotes liver fibrosis by producing TGF-β1, which mediates hepatic stellate cell activation and collagen expression [17]. However, the role of macrophage MERTK in efferocytosis and pulmonary fibrosis remains unknown. Here, we show that MERTK expression is increased in lung macrophages from IPF patients and bleomycin-induced pulmonary fibrosis mice compared with controls. Furthermore, MERTK-overexpressing macrophages promote TGF-β expression in macrophages and collagen expression in lung fibroblasts. In addition, macrophage efferocytosis downregulates MERTK in macrophages, thereby attenuating the profibrotic effects of MERTK and forming a negative regulatory loop. In pulmonary fibrosis, this negative regulation loop is defective.

## Materials and methods

### Collection of human lung tissues

This study was reviewed and approved by the Ethics Committee of The First Affiliated Hospital of Guangzhou Medical University. Tissues from explanted IPF lungs and healthy donor lungs were collected and used for isolation of lung macrophages. The IPF patients were diagnosed according to the 2018 ATS/ERS/JRS/ALAT guideline diagnostic criteria [18].

### Bleomycin (BLM)-induced pulmonary fibrosis mouse model

Male C57BL/6J mice (8–9 weeks old) were purchased from Hua Fu Kang Company (Beijing, China). The mouse model of pulmonary fibrosis was established as previously described [19, 20]. Briefly, BLM (2 mg/kg in 50 μL saline, Hanhui Pharmaceuticals, China) or an equal amount of saline was intratracheally administered to the BLM group or control group, respectively. Twenty-one days after airway administration, the mice were sacrificed, and the BALF and lungs were harvested for further analysis. This animal study was reviewed and approved by the Institutional Animal Care and Use Committee (IACUC) of Guangzhou Medical University.

### Histological evaluation and lung fibrosis scoring

The mouse lung tissue was fixed in 4% paraformaldehyde for 24 h, embedded in paraffin and cut into 3–5 μm slices. After paraffin was removed, the lung tissue slices were stained with haematoxylin and eosin (H&E) or Masson’s trichrome (Solarbio Life Science, Beijing, China) following the manufacturer’s instructions. The stained sections were digitally scanned using a digital pathological section scanner (PRECICE500B, Beijing, China). The degree of pulmonary fibrosis in histological samples was assessed by the Ashcroft score of Masson’s trichrome staining [21].

### Cell culture

A549 (human lung adenocarcinoma cell line, ATCC, USA), HLF (human lung fibroblast cell line, ATCC) and 293 T (human kidney epithelial cell line, ATCC) cells were grown in Dulbecco’s modified Eagle’s medium (DMEM, Gibco, USA) containing 10% foetal bovine serum (FBS, ExCell Bio, Shanghai, China) and 1% penicillin‒streptomycin solution. THP-1 cells (human monocyte cell line, ATCC) and MLE12 cells (mouse lung epithelial cell line, ATCC) were grown in RPMI-1640 (Gibco, USA) supplemented with 10% FBS and 1% penicillin‒streptomycin solution. All of the above cells were cultured at 37 °C in a humidified incubator with 5% CO_2_ (Thermo Scientific, Series II Water Jacket). THP-1 cells were differentiated into macrophages using 200 μg/mL phorbol 12-myristate 13-acetate (PMA, Abcam, USA).

### Isolation of human lung macrophages and mouse lung macrophages

Human lung macrophages and mouse lung macrophages were isolated as previously described [22, 23]. Briefly, in a cell culture dish containing Dulbecco’s phosphate buffered saline (DPBS, BasalMedia, Shanghai, China), human lung tissues were cut into small pieces, which were gently shaken and then removed. The liquid in the dish was a cell suspension containing human lung macrophages. Mouse lung macrophages were obtained from bronchoalveolar lavage fluid (BALF). For a single sample, the cell suspension was collected into a centrifuge tube and centrifuged at 700×*g* and 4 °C for 5 min. The supernatant was discarded, and the cell pellet was resuspended in erythrocyte lysate (Boster, Wuhan, China) at room temperature (RT) for 5 min and then centrifuged at 700×*g* and 4 °C for 5 min. The supernatant was then discarded, and the cell pellet was resuspended in RPMI-1640 and added to a cell culture plate. The cells were allowed to adhere to the plate for 1–2 h at 37 °C in a humidified incubator with 5% CO_2_ (Series II Water Jacket, Thermo Scientific, USA). Nonadherent cells were removed by gently washing three times with warm PBS. Other irrelevant adherent cells, such as lung epithelia, were removed by digestion with Accutase (StemPro™ Accutase™ Cell Dissociation Reagent, Gibco). Eventually, greater than 90% of the cultured cells were macrophages.

### Cell transfection

The primers for the human MERTK gene (NCBI NM_006343) were as follows: 5′- CCGCTCGAGATGGGGCCGGCCCCGCTGCCGCTG-3′(forward) and 5′- CCGGAATTCTCACATCAGGACTTCTGAGCC -3′ (reverse). A human MERTK cDNA clone (Youbio, Changsha, China) was used as a template to amplify the coding DNA sequence of MERTK, which was cloned into the pLVX-Puro vector to construct the pLVX-Puro-MERTK plasmid (Additional file 1**:** Fig. S3A-B). To obtain virus particles, the pLVX-Puro-MERTK plasmid was cotransfected with pMD2G and psPAX2 plasmids into 293 T cells using Lipofectamine 2000 (Invitrogen, USA). Viral particles were obtained 48 h after transfection and presented in the supernatant of 293 T cells. The blank pLVX-Puro plasmid (vector) was used as a negative control for overexpression experiments. After treatment with NATE™ (Nucleic Acid Transfection Enhancer, Invitrogen) for 30 min, THP-1 cells were cultured in the supernatant of 293 T cells containing viral particles (prefiltered with a 0.45 μm filter membrane). After 48 h, the culture medium was removed by centrifugation, and the successfully infected THP-1 cells were screened with 1 μg/ml puromycin for at least 48 h.

### Induction and quantification of cell apoptosis

To induce apoptosis, A549 and MLE12 cells were exposed to UV radiation (253.7 nm, 135 μW/cm^2^) for 1 h and then incubated in a 37 °C incubator for 20 h. Cell apoptosis was confirmed by flow cytometry using an apoptosis assay detection kit containing Annexin V and 7-AAD (marker for apoptosis and necrosis, respectively, BD, USA). The ratio of apoptosis was determined by the positive rate of Annexin V.

### Macrophage efferocytosis assay

Macrophage efferocytosis assays were performed as previously described [24–26]. Briefly, apoptotic epithelial cells (with an apoptotic rate greater than 70%) were prepared (Additional file 1**:** Fig. S1) and labelled with CFSE (5,6-carboxyfluorescein diacetate, succinimidyl ester, Cell Division Tracker Kit, BioLegend, USA). Apoptotic A549 cells were cocultured with primary human lung macrophages or THP-1 cells, while apoptotic MLE12 cells were cocultured with primary mouse lung macrophages. After coculturing with sufficient CFSE-labelled apoptotic epithelial cells (apoptotic cells: Mø > 10:1), macrophages were labelled with anti-human CD68-APC (1:40 dilution for flow cytometry and 1:25 dilution for immunofluorescence, BioLegend) or anti-mouse F4/80-PE (1:50 dilution for flow cytometry and 1:25 dilution for immunofluorescence, BioLegend). The ratio and capacity of macrophage efferocytosis were analysed by fluorescence microscopy (Leica DM6 FS, Germany) and flow cytometry (FACS Verse, BD), respectively, and determined by detecting the CFSE-positive ratio and mean fluorescence intensity (MFI) of CFSE in macrophages, respectively.

### Fluorescence staining and quantification of lung macrophage MERTK

Lung sections of IPF patients and healthy controls (HCs) were permeabilized with 0.25% Triton X-100 in PBS. After blocking with 10% goat serum and Fc receptor blocking solution (1:25 dilution, BioLegend), lung sections were incubated with anti-Mertk antibody (1:50 dilution, Cell Signaling Technology) overnight at 4 °C, followed by staining with FITC-conjugated secondary antibody (1:500 dilution, Abcam) for 1 h at room temperature. Lung macrophages were labelled with PE anti-human CD68 antibody (1:25 dilution, BioLegend) for 30 min at 4 °C. DAPI was used as a nuclear counterstain. Images of Mertk in CD68^+^ cells were acquired with an Olympus IX83 microscope. The fluorescence intensity of MERTK in CD68^+^ cells was analysed by ImageJ software (NIH, Bethesda, MA, USA).

### RNA extraction and quantitative real-time PCR (qRT‒PCR)

Total RNA from lung tissues or cells was extracted using Nuclezol RNA Isolation Plus Reagent (Macherey–Nagel, Germany). Afterwards, reverse transcription reactions were performed on the extracted RNA using a Hiffair^®^ III Reverse Transcriptase Kit (YEASEN, Shanghai, China) according to the manufacturer’s instructions. Real-time RT‒PCR amplification was performed using Hieff UNICON^®^ qPCR SYBR Green Master Mix (YEASEN, Shanghai, China) on a QuantStudio 5 Real-Time PCR System (Thermo Fisher Scientific, USA). Relative mRNA expression levels were calculated using the 2^−ΔΔCt^ method and normalized to GAPDH or β-actin. The primer sequences are listed in Table 1.

**Table 1:** Primer sequences used in this study

Gene	Forward Primer (5′–3′)	Reverse Primer (5′–3′)
Human_MertK	CTCTGGCGTAGAGCTATCACT	AGGCTGGGTTGGTGAAAACA
Human_CD36	GGCTGTGACCGGAACTGTG	AGGTCTCCAACTGGCATTAGAA
Human_Rac1	ATGTCCGTGCAAAGTGGTATC	CTCGGATCGCTTCGTCAAACA
Human_Axl	GTGGGCAACCCAGGGAATATC	GTACTGTCCCGTGTCGGAAAG
Human_15-LO	GGGCAAGGAGACAGAACTCAA	CAGCGGTAACAAGGGAACCT
Human_Fibronectin	CGGTGGCTGTCAGTCAAAG	AAACCTCGGCTTCCTCCATAA
Human_Col1a1	CCCGTTGGCAAAGATGGTAG	ACCTTGGCTACCCTGAGAAC
Human_Col3a1	GGAGCTGGCTACTTCTCGC	GGGAACATCCTCCTTCAACAG
Human_α-SMA	GCTGGTGATGATGCTCCCA	GCCCATTCCAACCATTACTCC
Human_GAPDH	AACGACCCCTTCATTGACCT	CATTCTCGGCCTTGACTGTG
Mouse_MertK	CAGGGCCTTTACCAGGGAGA	TGTGTGCTGGATGTGATCTTC
Mouse_CD36	ATGGGCTGTGATCGGAACTG	TTTGCCACGTCATCTGGGTTT
Mouse_Rac1	GAGACGGAGCTGTTGGTAAAA	ATAGGCCCAGATTCACTGGTT
Mouse_15-LO	GGCTCCAACAACGAGGTCTAC	CCCAAGGTATTCTGACACATCC
Mouse_Col1a1	GCTCCTCTTAGGGGCCACT	CCACGTCTCACCATTGGGG
Mouse_Col3a1	CTGTAACATGGAAACTGGGGAAA	CCATAGCTGAACTGAAAACCACC
Mouse_β-actin	GGCTGTATTCCCCTCCATCG	CCAGTTGGTAACAATGCCATGT

### Western blotting

The mouse lung tissues and cells were lysed in RIPA lysis buffer (Beyotime, Shanghai, China) containing protease and phosphatase inhibitors (Beyotime, Shanghai, China) and incubated on ice for 30 min. After centrifugation at 12,000×*g* at 4 °C for 20 min, the supernatant was collected. The protein concentration was determined using a BCA protein assay kit (Thermo Scientific). Equal amounts of protein in cell lysates were separated by SDS‒PAGE and transferred to polyvinylidene fluoride (PVDF) membranes (Millipore). The membranes were blocked with 5% w/v skim milk for 1 h and then incubated with primary antibodies against MERTK (1:1000, Cell Signaling Technology), TGF-β (1:1000, Cell Signaling Technology), α-SMA (1:1000 dilution, Abcam), type I collagen (1:1000 dilution, Abcam), type III collagen (1:1000 dilution, Abcam), fibronectin (1:1000 dilution, Santa Cruz), and β-actin (1:10,000 dilution, Cell Signaling Technology) at 4 °C overnight. Afterwards, the blots were incubated with peroxidase-conjugated secondary anti-mouse or anti-rabbit IgG for 1 h at RT. The blots were analysed using the StarSignal Plus Chemiluminescent assay kit (GenStar, Beijing, China) and developed using a TANON 5200 automated chemiluminescence imaging analysis system (Tanon, Shanghai, China). β-Actin was used to normalize sample loading, and the intensities of the bands were quantified using ImageJ software.

### Statistical analysis

Data are presented as the mean ± SEM, and each experiment was repeated at least three times. T tests or Mann‒Whitney tests were applied to two-group analyses, while ANOVA, Kruskal‒Wallis tests or post hoc analysis were used when comparing more than two groups. All analyses were performed using GraphPad Prism 8 software. *p < 0.05; **p < 0.01; ***p < 0.001; ****p < 0.0001; n.s., not significant. The difference between groups was statistically significant when p < 0.05.

## Results

### Lung macrophage efferocytosis function in IPF patients is not enhanced by elevated MERTK expression

**Fig. 1:**
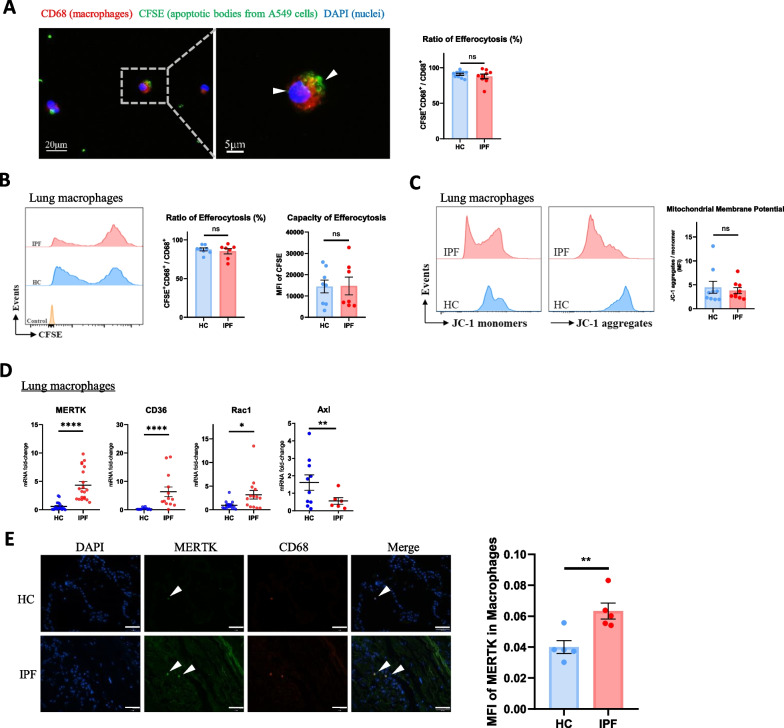
Lung macrophage efferocytosis function in IPF patients is not enhanced by elevated MERTK expression. **A** Efferocytosis (indicated by white arrowheads) of human lung macrophages under fluorescence microscope and ratio of macrophages that recognize and phagocytize CFSE + apoptotic bodies from A549 cells (HC, n = 10; IPF, n = 9). **B** Flow cytometry analysis of human lung macrophage efferocytosis. Lung macrophages were pre-gated on CD68 + area, ratio and capacity of macrophage efferocytosis were measured by CFSE-positive ratio (CFSE + /CFSE + CD68 +) and mean fluorescence intensity (MFI) of CFSE, respectively (HC, n = 8; IPF, n = 7). **C** Flow cytometry analysis of mitochondrial membrane potential of human lung macrophages. Lung macrophages were pre-gated on CD68 + area, and their mean fluorescence intensities of JC-1 monomers (FITC) and aggregates (PE) were analyzed by flow cytometry (HC, n = 9; IPF, n = 9). **D** The mRNA expression of MERTK, CD36, Rac1 and Axl in human lung macrophages was detected by qRT-PCR (n = 6–20 samples per group). **E** Representative images and MFI of MERTK immunostaining in macrophages from lung sections of IPF patients and healthy controls (n = 5). Macrophages were indicated by white arrowheads. Scale bar = 50 μm. Data were presented as mean ± SEM. T-tests or Mann–Whitney tests were used for two-group (HC and IPF) comparions. *p < 0.05; **p < 0.01; ****p < 0.0001; n.s., not significant

To compare the function of lung macrophage efferocytosis in healthy controls (HCs) and IPF patients, we isolated lung macrophages from human lung tissue. The isolated human lung macrophages were then cocultured with sufficient CFSE-labelled apoptotic A549 cells (apoptotic cells: Mø > 10:1) [24], labelled with anti-CD68-APC, and analysed by fluorescence microscopy or flow cytometry. The function of efferocytosis is determined by the ratio and capacity of macrophage efferocytosis. The ratio of efferocytosis is the percentage of macrophages that have the ability to “eat” apoptotic cells, while the capacity of efferocytosis is measured as the average amount of apoptotic cells “eaten” by each individual macrophage; these indices were determined by the CFSE-positive ratio and mean fluorescence intensity (MFI) of CFSE detected in macrophages, respectively [13, 24, 26]. We found that the ratio and capacity of efferocytosis in lung macrophages from healthy controls and IPF patients were not significantly different (Fig. 1A, B), suggesting that lung macrophages from these two sources have an equal ability to “eat” apoptotic cells.

As mitochondrial membrane potential within phagocytes is a critical regulator of efferocytosis, we examined the mitochondrial membrane potential of lung macrophages [13, 27]. After staining with JC-1 dye, lung macrophages were analysed by fluorescence microscopy and flow cytometry, and mitochondrial membrane potential was quantified by the MFI ratio of JC-1 aggregates to JC-1 monomers. As shown in Fig. 1C, the mitochondrial membrane potential of HC and IPF lung macrophages was not different, which may explain the lack of a significant difference in efferocytosis. We also detected the mRNA expression of several efferocytosis-related genes in macrophages. The mRNA expression levels of MERTK, CD36 (both macrophage apoptotic cell recognition receptors) and Rac1 (an indicator of cytoskeletal rearrangement for phagocytosis) were increased in IPF patients, while the mRNA level of Axl (a receptor tyrosine kinase from the TAM family) was not significantly different between the two groups (Fig. 1D). We also examined the protein level of macrophage MERTK in lung sections from IPF patients and healthy controls by immunostaining. As shown in Fig. 1E, MERTK was upregulated in lung macrophages from IPF patients.

### Efferocytosis function is impaired while MERTK is elevated in lung macrophages from mice with bleomycin-induced pulmonary fibrosis

To investigate efferocytosis in mice with pulmonary fibrosis, we first induced pulmonary fibrosis in mice by intratracheal administration of bleomycin (BLM) as previously described [20], and the control group was administered the same amount of normal saline (NS). Mice were sacrificed on Day 21 after intratracheal administration of BLM or NS. H&E and Masson’s trichrome staining showed inflammatory cell infiltration, thickened alveolar septa, and collagen accumulation in the lung tissue of BLM mice. The degree of pulmonary fibrosis was assessed by the Ashcroft score of Masson’s trichrome staining in histological sections [21]. The pathology results showed obvious pulmonary fibrosis in the lung tissue of BLM mice (Additional file 1**:** Fig. S2A). In addition, collagen expression and hydroxyproline content in the lung tissue of BLM mice were markedly increased, suggesting a higher collagen content (Additional file 1**:** Fig. S2B-C).

**Fig. 2:**
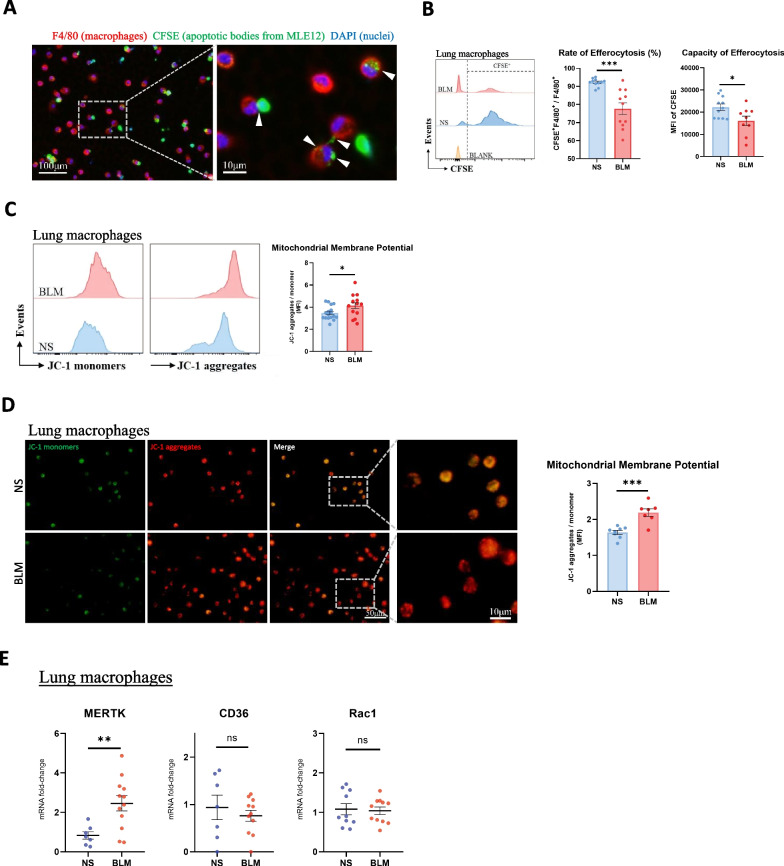
Efferocytosis function is impaired while MERTK is elevated in lung macrophages from mice with bleomycin-induced pulmonary fibrosis. **A** Efferocytosis of mouse lung macrophages under fluorescence microscope. As indicated by white arrowheads, macrophages (F4/80 + , red) recognize and phagocytize apoptotic bodies of MLE12 (CFSE + , green). **B** Flow cytometry analysis of mouse lung macrophage efferocytosis. Lung macrophages were pre-gated on F4/80 + area, ratio and capacity of macrophage efferocytosis were measured by CFSE-positive ratio (CFSE + /[CFSE + F4/80 +]) and MFI of CFSE, respectively (NS, n = 11; BLM, n = 9; BLANK: macrophages were cultured without addition of apoptotic cells). **C** Flow cytometry analysis of mitochondrial membrane potential of mouse lung macrophages. Lung macrophages were pre-gated on F4/80 + area, and the mean fluorescence intensities of JC-1 monomers (FITC) and aggregates (PE) were analyzed by flow cytometry (NS, n = 17; BLM, n = 14). **D** After staining with JC-1, the fluorescence staining of lung macrophages was observed under a fluorescence microscope. Mitochondrial membrane potential was semi-quantified by the ratio of the mean fluorescence intensity of JC-1 aggregates (red) to JC-1 monomers (green) (NS, n = 8; BLM, n = 7). **E** The mRNA expression of MERTK, CD36 and Rac1 in mouse lung macrophages was detected by qRT-PCR (n = 7–12 mice per group). Data were presented as mean ± SEM. T-tests or Mann–Whitney tests were used for two-group (NS and BLM) comparsions. *p < 0.05; **p < 0.01; ***p < 0.001; n.s., not significant

To compare the efferocytosis function of lung macrophages in NS and BLM mice, we isolated lung macrophages from mouse bronchoalveolar lavage fluid (BALF) and cocultured them with sufficient CFSE-labelled apoptotic MLE12 cells (apoptotic cells: Mø > 10:1). Macrophages were then labelled with anti-F4/80-APC and analysed by fluorescence microscopy or flow cytometry. As shown in Fig. 2A, B, the ratio and capacity of efferocytosis in lung macrophages from BLM mice were both lower than those from control mice, suggesting impaired efferocytosis function of lung macrophages in BLM mice. To explore the underlying mechanism, we examined the mitochondrial membrane potential and mRNA expression of certain efferocytosis-related genes in macrophages. We found an increased mitochondrial membrane potential of lung macrophages from BLM mice (Fig. 2C, D), which indicated that these lung macrophages may exhibit reduced continued clearance of apoptotic cells. In addition, the mRNA expression level of MERTK in the lung macrophages of BLM mice was elevated, while their CD36 and Rac1 expression levels were not significantly different from those of the control mice (Fig. 2E). The above results indicated that the impaired efferocytosis function of lung macrophages in BLM mice may be due to the increased mitochondrial membrane potential.

Collectively, these data showed that the expression of MERTK was increased, but the efferocytosis function in lung macrophages from both IPF patients and BLM mice was not enhanced with elevated MERTK expression.

### MERTK-overexpressing macrophages enhance efferocytosis function through the Ucp2/mitochondrial signalling pathway

Previous studies have shown that MERTK promotes function of macrophage efferocytosis; however, the underlying mechanism remains elusive [26]. We explored in detail how macrophage MERTK promotes efferocytosis function. The MERTK coding sequence was cloned into the pLVX-Puro vector and transfected into THP-1 cells. As shown in Additional file 1**:** Fig. S3C, MERTK expression at the mRNA and protein levels was significantly increased in these THP-1 cells compared to THP-1 cells transfected with blank pLVX-Puro (vector).

**Fig. 3:**
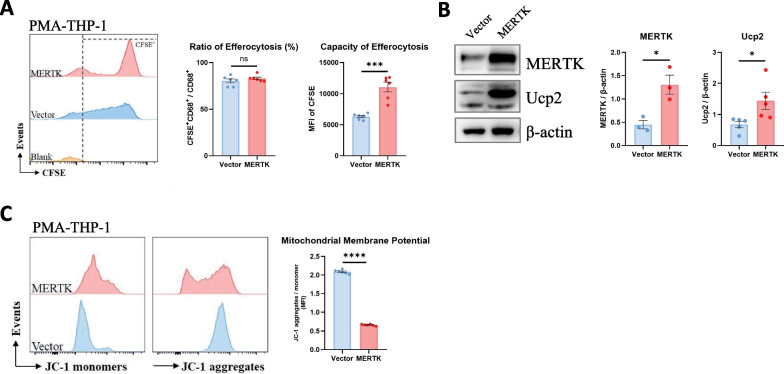
MERTK-overexpressing macrophages enhance efferocytosis function through the Ucp2/mitochondrial signalling pathway. **A** After coculturing apoptotic cells with vector- and MERTK-macrophages, the ratio and capacity of macrophage efferocytosis were detected by flow cytometry. Macrophages were pre-gated on CD68 + area, ratio and capacity of macrophage efferocytosis were measured by CFSE-positive ratio (CFSE + /CFSE + CD68 +) and MFI of CFSE, respectively (n = 6). **B** The protein expression of MERTK and Ucp2 in lung macrophages was detected by WB (n = 3–5 samples per group). **C** Mitochondrial membrane potential of vector- and MERTK-macrophages was measured by flow cytometry (n = 6). Data were presented as mean ± SEM. T-tests or Mann–Whitney tests were used for two-group (Vector and MERTK) comparsions. *p < 0.05; ***p < 0.001; ****p < 0.0001; n.s., not significant

We incubated vector- and MERTK-overexpressing macrophages with sufficient apoptotic cells, and flow cytometry analysis revealed that the ratio of efferocytosis in macrophages did not differ between the two groups, while the capacity of efferocytosis by each individual macrophage increased in MERTK-overexpressing macrophages, indicating that macrophage MERTK enhances efferocytosis function (Fig. 3A).

Mitochondrial uncoupling protein 2 (Ucp2) has been shown to regulate ions and protons across the mitochondrial membrane, thereby lowering the mitochondrial membrane potential and enhancing the capacity of phagocyte efferocytosis [13, 27]. We then wondered whether MERTK enhances macrophage efferocytosis function through the Ucp2/mitochondrial pathway. To this end, we examined Ucp2 expression in vector- and MERTK-overexpressing macrophages and found that MERTK promotes Ucp2 expression in macrophages (Fig. 3B). In addition, we detected the mitochondrial membrane potential of macrophages after JC-1 staining by flow cytometry and found decreased mitochondrial membrane potential in MERTK-overexpressing macrophages (Fig. 3C). Together, these data indicated that macrophage MERTK promotes efferocytosis by upregulating Ucp2, which decreases the mitochondrial membrane potential.

### MERTK-overexpressing macrophages exhibit profibrotic effects, and efferocytosis inhibits these profibrotic effects

**Fig. 4:**
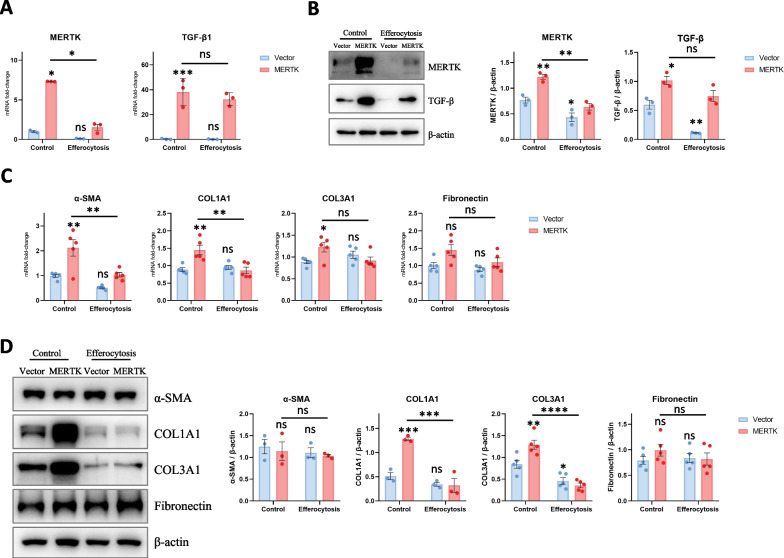
MERTK-overexpressing macrophages exhibit profibrotic effects, and efferocytosis inhibits these profibrotic effects. **A** After coculturing apoptotic cells with vector- and MERTK-macrophages, the expression of MERTK and TGF-β1 in macrophages was detected by qRT-PCR (n = 3). **B** After coculturing apoptotic cells with vector- and MERTK-macrophages, the expression of MERTK and TGF-β in macrophages was detected by WB (n = 3). **C** Human lung fibroblasts (HLFs) were incubated with the conditioned medium from vector- and MERTK-macrophages, the mRNA expression of α-SMA, COL1A1, COL3A1 and fibronectin in lung fibroblasts was detected by qRT-PCR (n = 5). **D** HLFs were incubated with the conditioned medium from vector- and MERTK-macrophages, the protein expression of α-SMA, COL1A1, COL3A1 and fibronectin in lung fibroblasts was detected by WB (n = 3–5 samples per group). Data were presented as mean ± SEM. Two-way ANOVA with Tukey’s multiple comparisons test was used for analysis. *p < 0.05; **p < 0.01; ***p < 0.001; ****p < 0.0001; n.s., not significant. The asterisk above each bar denotes significant difference between this group and Control-Vector group, while the asterisk on the horizontal line denotes significant difference between control-MERTK group and efferocytosis-MERTK group

We further investigated whether macrophage MERTK exhibits pro- or antifibrotic effects. MERTK-overexpressing THP-1 cells and control cells were induced to differentiate into macrophages by incubation with PMA, and then the expression levels of MERTK and TGF-β (a well-known profibrotic factor) in macrophages were detected. The results suggested that macrophage MERTK promotes TGF-β expression (Fig. 4A, B), consistent with previous studies [17]. As lung fibroblasts are major effector cells in pulmonary fibrosis, we also explored whether MERTK-overexpressing macrophages promote the activation of and collagen secretion by lung fibroblasts. The culture medium of vector- and MERTK-macrophages was collected and filtered as conditioned medium to treat human lung fibroblasts (HLFs). After treatment, we detected the expression of α-SMA, collagen I, and collagen III in HLF. The data showed that conditioned medium from MERTK-overexpressing macrophages enhanced collagen expression in HLFs (Fig. 4C, D). Together, these results revealed that macrophage MERTK promotes the expression of profibrotic factors (such as TGF-β) in macrophages and collagen expression in lung fibroblasts; thus, exhibiting a profibrotic role.

MERTK is a macrophage membrane receptor that recognizes apoptotic cells in efferocytosis [11, 28]. We next wondered whether the profibrotic effect of macrophage MERTK is dependent on efferocytosis. Therefore, we induced efferocytosis of vector- and MERTK-macrophages by coculturing them with sufficient apoptotic epithelial cells (UV-A549) and examined the expression of MERTK and TGF-β in macrophages. As shown in Fig. 4A, B, efferocytosis decreased MERTK expression in both vector- and MERTK-macrophages and decreased TGF-β expression in vector-macrophages, indicating that the profibrotic effect of macrophage MERTK is independent of efferocytosis and that macrophage efferocytosis exhibits an antifibrotic effect. In addition, we cultured human lung fibroblasts with conditioned medium from macrophages to further confirm whether macrophage efferocytosis exhibits an antifibrotic effect. The data showed that conditioned medium from the efferocytosis group downregulated collagen expression in human lung fibroblasts (Fig. 4C, D). These findings suggested that macrophage MERTK exhibits a profibrotic effect, while macrophage efferocytosis inhibits the profibrotic effect of MERTK. This may be a protective mechanism that prevents macrophages from entering a profibrotic state.

## Discussion

**Fig. 5:**
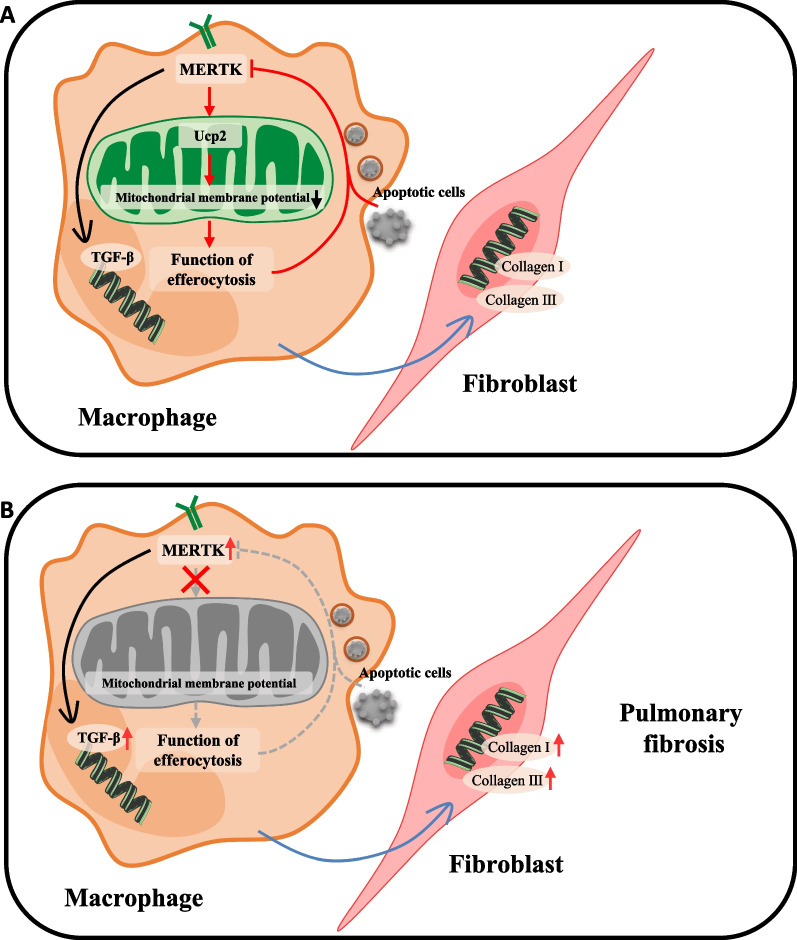
Elevated macrophage MERTK exhibits pro-fibrotic effects and results in defective regulation of efferocytosis function in pulmonary fibrosis. **A** Macrophage MERTK exhibits profibrotic effects via increasing TGF-β expression in macrophages and collagen expression in lung fibroblasts. Macrophage MERTK enhances efferocytosis function by up-regulating Ucp2, which can lower macrophage mitochondrial membrane potential. Macrophage efferocytosis attenuates the profibrotic effect of MERTK by downregulating MERTK, forming a negative regulatory loop. **B** In pulmonary fibrosis, MERTK expression is abnormally increased in lung macrophages and the above negative regulatory loop is defective

IPF and BLM-induced pulmonary fibrosis are characterized by the accumulation of damaged and apoptotic alveolar epithelial cells [3, 6, 29]. Our study showed that lung macrophages from both IPF patients and BLM mice have a common feature of high MERTK expression. We also found that macrophage MERTK plays a profibrotic role and that macrophage efferocytosis downregulates MERTK expression in macrophages, thereby attenuating the profibrotic effect of MERTK (Fig. 5A). In pulmonary fibrosis, this regulatory mechanism is defective (Fig. 5B).

A relationship between efferocytosis and pulmonary fibrosis has been suggested by previous studies. Morimoto et al. showed reduced efferocytosis by alveolar macrophages in IPF patients compared to subjects with other interstitial pneumonitis (such as nonspecific interstitial pneumonitis, cryptogenic organizing pneumonia and eosinophilic pneumonia), but they did not explore the differences in lung macrophage efferocytosis function between IPF patients and healthy subjects [8]. Here, we isolated lung macrophages and cocultured them with excess apoptotic cells in vitro. No remarkable difference in macrophage efferocytosis function was found between IPF patients and healthy controls, indicating that in the presence of excess apoptotic cells in the extracellular environment, the macrophages of IPF patients and healthy controls have equal ability to "eat" apoptotic cells. In BLM-induced pulmonary fibrosis mice, the efferocytosis function of lung macrophages was impaired but not completely lost. Our results showed that macrophage efferocytosis is largely related to mitochondrial membrane potential. A lower mitochondrial membrane potential indicates a higher capability of macrophages to clear apoptotic cells [13, 27, 30]. We also detected elevated 15-LO expression in lung macrophages from both IPF patients and mice with BLM-induced pulmonary fibrosis (Additional file 1**:** Fig. S4), suggesting an enhanced macrophage efferocytosis activity in pulmonary fibrosis. It is not difficult to imagine that with increasing apoptotic cells in the alveoli, macrophage efferocytosis is more active.

MERTK and mitochondrial dynamics play crucial roles in efferocytosis [13, 27, 28, 31–34]. Our study discovered that macrophage MERTK enhances efferocytosis function via the Ucp2/mitochondrial signalling pathway and that efferocytosis function inhibits the expression of MERTK, forming a negative regulatory loop. However, in pulmonary fibrosis, although MERTK expression is increased, mitochondrial membrane potential and efferocytosis function are not enhanced, leading to defective negative regulation. It has been shown that macrophage MERTK enhances TGF-β1 expression, promoting liver fibrosis [17, 32]. In IPF patients, macrophage MERTK expression is increased in lung fibrotic lesions compared to nonfibrotic lesions [16]. Here, we not only detected markedly elevated MERTK expression in lung macrophages from IPF patients and bleomycin mice but also confirmed that elevated MERTK expression in macrophages promotes collagen expression in lung fibroblasts, indicating a profibrotic role of macrophage MERTK. This study has limitations. Few macrophages were isolated from either human lung tissue or mouse BALF, and they hardly proliferated in vitro, making it difficult to examine protein expression in lung macrophages. In addition, even though many efforts have been made in the research of efferocytosis [24, 25, 35, 36], there is still a lack of unified and stable assays to assess efferocytosis function. Before testing the efferocytosis function of phagocytes, researchers need to prepare phagocytes (apoptotic cells/apoptotic bodies), so the types of apoptotic cells, the methods of inducing apoptosis and the apoptosis rate may vary in different studies.

## Conclusions

In summary, we found elevated MERTK expression in lung macrophages from IPF patients and mice with bleomycin-induced pulmonary fibrosis. Moreover, our study reveals a previously unrecognized profibrotic effect of elevated macrophage MERTK in pulmonary fibrosis and defective regulation of efferocytosis function as a result of that elevation, suggesting that targeting MERTK in macrophages may help to attenuate pulmonary fibrosis.

## Supplementary Information

**Additional file 1: Figure S1**. Flow cytometry analysis of apoptosis. Apoptosis of A549 and MLE12 cells was induced by exposure to UV radiation for 1 h and incubated at 37 °C in an incubator for 20 h. Cell apoptosis was confirmed by flow cytometry using an apoptosis assay detection kit containing Annexin V and 7-AAD. The ratio of apoptosis was quantified by the positive rate of Annexin V. Data were presented as mean ± SEM. Two-way ANOVA with Tukey’s multiple comparisons test were used for two-groupcomparsions. **p < 0.01; ****p < 0.0001.** Figure S2**. Bleomycin-induced pulmonary fibrosis in mice. H&E and Masson’s trichrome staining of mouse lung histopathological sections. The degree of pulmonary fibrosis was assessed by the Ashcroft score. A Hydroxyproline content in lung tissue of the mice. B mRNA expression of COL1A1 and COL3A1 in lung tissue of the mice. Data were presented as mean ± SEM. T-tests or Mann–Whitney tests were used for two-groupcomparsions. **p < 0.01; ***p < 0.001; ****p < 0.0001.** Figure S3**. Sequencing analysis of overexpression vector and verification after cell transfection. A Sequencing results of overexpression vectors analyzed by snapgene. B The results of the protein prediction based on the sequence were analyzed by Blastxof NCBI. The best matching result is MERTK. C After transfection, MERTK expression in THP-1 cells was detected by qRT-PCR and WB. Data were presented as mean ± SEM. One-way ANOVA with Dunnett’s multiple comparisons test was used for analysis. **p < 0.01; ****p < 0.0001; n.s., not significant. The asterisk above each bar denotes significant difference between this group and Control group.** Figure S4**. Efferocytosis activity is increased in pulmonary fibrosis. The mRNA expression of 15-LO in human lung macrophages and mouse lung macrophages was detected by qRT-PCR. Data were presented as mean ± SEM. Mann–Whitney tests were used for two-groupcomparsions. *p < 0.05; **p < 0.01.

## Data Availability

All data generated during this study are included in this article.
