# Supplementary material for: Elevated expression of macrophage MERTK exhibits profibrotic effects and results in defective regulation of efferocytosis function in pulmonary fibrosis
Source: Respir Res. 2023 Apr 29;24:118. doi: 10.1186/s12931-023-02424-3 (PMC10148433; doi:10.1186/s12931-023-02424-3)

Supplementary Figure 1

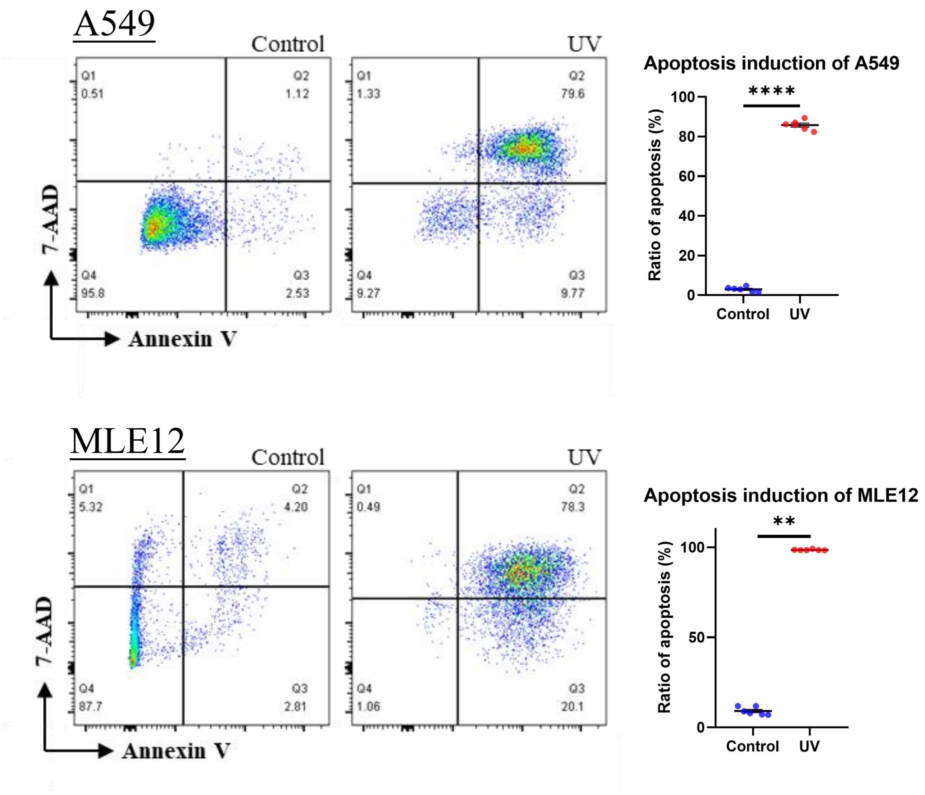

Supplementary Figure 2

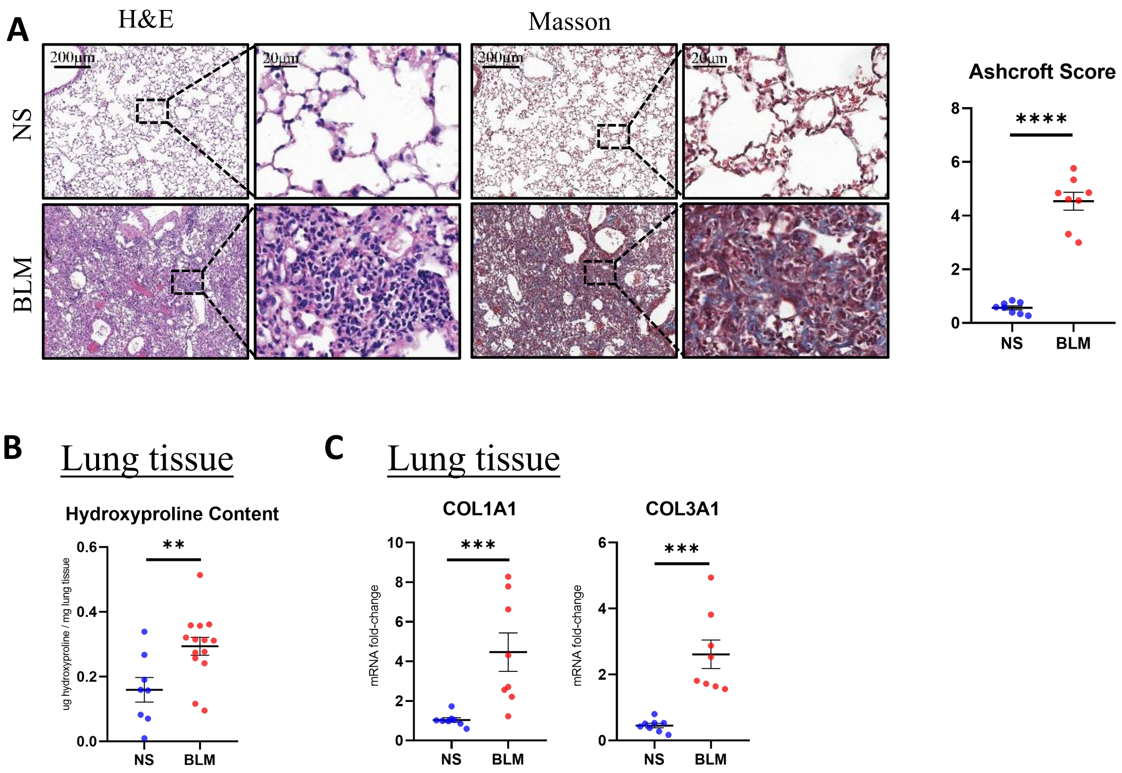

Supplementary Figure 3

A

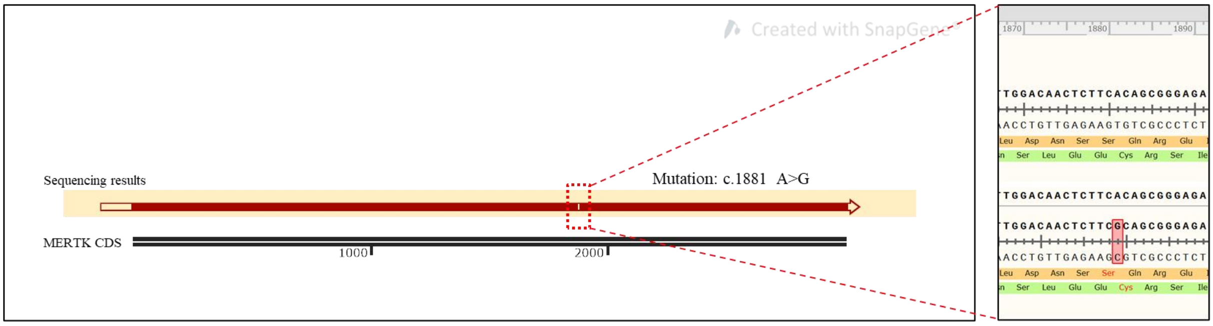

B

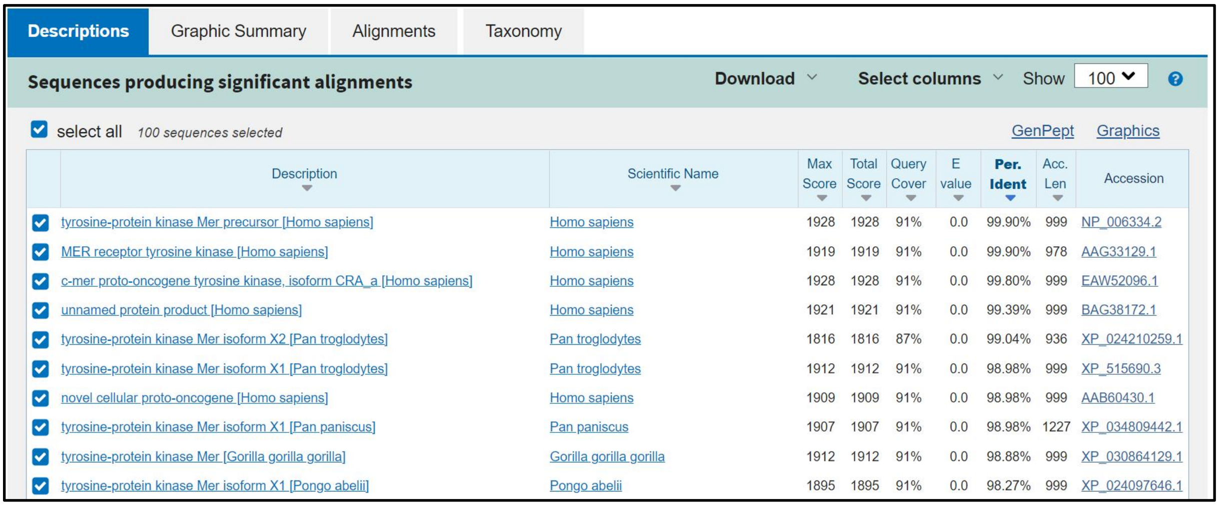

Descriptions | Graphic Summary | Alignments | Taxonomy

Sequences producing significant alignments — Download — Select columns — Show 100

select all — *100 sequences selected* — GenPept — Graphics

Description	Scientific Name	Max Score	Total Score	Query Cover	E value	Per. Ident	Acc. Len	Accession
tyrosine-protein kinase Mer precursor [Homo sapiens]	Homo sapiens	1928	1928	91%	0.0	99.90%	999	NP_006334.2
MER receptor tyrosine kinase [Homo sapiens]	Homo sapiens	1919	1919	91%	0.0	99.90%	978	AAG33129.1
c-mer proto-oncogene tyrosine kinase, isoform CRA_a [Homo sapiens]	Homo sapiens	1928	1928	91%	0.0	99.80%	999	EAW52096.1
unnamed protein product [Homo sapiens]	Homo sapiens	1921	1921	91%	0.0	99.39%	999	BAG38172.1
tyrosine-protein kinase Mer isoform X2 [Pan troglodytes]	Pan troglodytes	1816	1816	87%	0.0	99.04%	936	XP_024210259.1
tyrosine-protein kinase Mer isoform X1 [Pan troglodytes]	Pan troglodytes	1912	1912	91%	0.0	98.98%	999	XP_515690.3
novel cellular proto-oncogene [Homo sapiens]	Homo sapiens	1909	1909	91%	0.0	98.98%	999	AAB60430.1
tyrosine-protein kinase Mer isoform X1 [Pan paniscus]	Pan paniscus	1907	1907	91%	0.0	98.98%	1227	XP_034809442.1
tyrosine-protein kinase Mer [Gorilla gorilla gorilla]	Gorilla gorilla gorilla	1912	1912	91%	0.0	98.88%	999	XP_030864129.1
tyrosine-protein kinase Mer isoform X1 [Pongo abelii]	Pongo abelii	1895	1895	91%	0.0	98.27%	999	XP_024097646.1

C

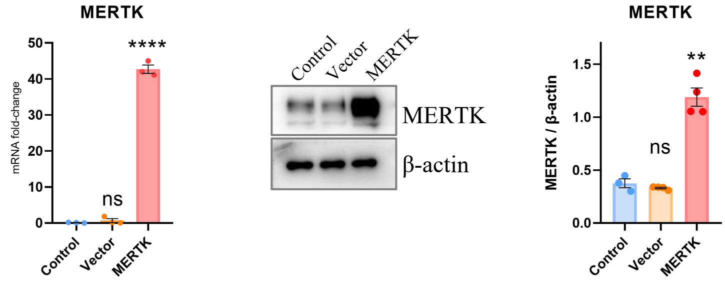

Supplementary Figure 4

A

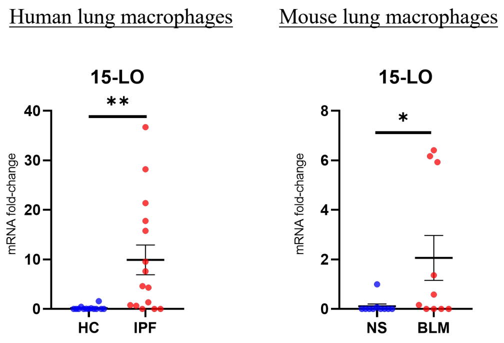

Supplement: Supplementary file 1 — Additional file 1: Figure S1. Flow cytometry analysis of apoptosis. Apoptosis of A549 and MLE12 cells was induced by exposure to UV radiation for 1 h and incubated at 37 °C in an incubator for 20 h. Cell apoptosis was confirmed by flow cytometry using an apoptosis assay detection kit containing Annexin V and 7-AAD. The ratio of apoptosis was quantified by the positive rate of Annexin V. Data were presented as mean ± SEM. Two-way ANOVA with Tukey’s multiple comparisons test were used for two-groupcomparsions. **p < 0.01; ****p < 0.0001. Figure S2. Bleomycin-induced pulmonary fibrosis in mice. H&E and Masson’s trichrome staining of mouse lung histopathological sections. The degree of pulmonary fibrosis was assessed by the Ashcroft score. A Hydroxyproline content in lung tissue of the mice. B mRNA expression of COL1A1 and COL3A1 in lung tissue of the mice. Data were presented as mean ± SEM. T-tests or Mann–Whitney tests were used for two-groupcomparsions. **p < 0.01; ***p < 0.001; ****p < 0.0001. Figure S3. Sequencing analysis of overexpression vector and verification after cell transfection. A Sequencing results of overexpression vectors analyzed by snapgene. B The results of the protein prediction based on the sequence were analyzed by Blastxof NCBI. The best matching result is MERTK. C After transfection, MERTK expression in THP-1 cells was detected by qRT-PCR and WB. Data were presented as mean ± SEM. One-way ANOVA with Dunnett’s multiple comparisons test was used for analysis. **p < 0.01; ****p < 0.0001; n.s., not significant. The asterisk above each bar denotes significant difference between this group and Control group. Figure S4. Efferocytosis activity is increased in pulmonary fibrosis. The mRNA expression of 15-LO in human lung macrophages and mouse lung macrophages was detected by qRT-PCR. Data were presented as mean ± SEM. Mann–Whitney tests were used for two-groupcomparsions. *p < 0.05; **p < 0.01. [file 12931_2023_2424_MOESM1_ESM.pdf]
